# Neural Signatures of Handgrip Fatigue in Type 1 Diabetic Men and Women

**DOI:** 10.3389/fnhum.2020.564969

**Published:** 2020-11-09

**Authors:** Oshin Tyagi, Yibo Zhu, Connor Johnson, Ranjana K. Mehta, Farzan Sasangohar, Madhav Erraguntla, Khalid Qaraqe

**Affiliations:** ^1^Department of Industrial and Systems Engineering, Texas A&M University, College Station, TX, United States; ^2^Department of Electrical and Computer Engineering, Texas A&M University at Qatar, Doha, Qatar

**Keywords:** motor performance, fNIRS, gender, functional connectivity, strength

## Abstract

Type 1 diabetes (T1D) is associated with reduced muscular strength and greater muscle fatigability. Along with changes in muscular mechanisms, T1D is also linked to structural changes in the brain. How the neurophysiological mechanisms underlying muscle fatigue is altered with T1D and sex related differences of these mechanisms are still not well investigated. The aim of this study was to determine the impact of T1D on the neural correlates of handgrip fatigue and examine sex and T1D related differences in neuromuscular performance parameters, neural activation and functional connectivity patterns between the motor regions of the brain. Forty-two adults, balanced by condition (healthy vs T1D) and sex (male vs female), and performed submaximal isometric handgrip contractions until voluntary exhaustion. Initial strength, endurance time, strength loss, force variability, and complexity measures were collected. Additionally, hemodynamic responses from motor-function related cortical regions, using functional near-infrared spectroscopy (fNIRS), were obtained. Overall, females exhibited lower initial strength (*p* < 0.0001), and greater strength loss (*p* = 0.023) than males. While initial strength was significantly lower in the T1D group (*p* = 0.012) compared to the healthy group, endurance times and strength loss were comparable between the two groups. Force complexity, measured as approximate entropy, was found to be lower throughout the experiment for the T1D group (*p* = 0.0378), indicating lower online motor adaptability. Although, T1D and healthy groups fatigued similarly, only the T1D group exhibited increased neural activation in the left (*p* = 0.095) and right (*p* = 0.072) supplementary motor areas (SMA) over time. A sex × condition × fatigue interaction effect (*p* = 0.044) showed that while increased activation was observed in both T1D females and healthy males from the Early to Middle phase, this was not observed in healthy females or T1D males. These findings demonstrate that T1D adults had lower adaptability to fatigue which they compensated for by increasing neural effort. This study highlights the importance of examining both neural and motor performance signatures when investigating the impact of chronic conditions on neuromuscular fatigue. Additionally, the findings have implications for developing intervention strategies for training, rehabilitation, and ergonomics considerations for individuals with chronic conditions.

## Introduction

Diabetes is growing at an alarming rate (Skyler and Oddo, 2002; Lam and LeRoith, 2012) and its cost in the United States surpassed $237 Billion in 2017 (American Diabetes Association, 2018). Individuals with diabetes are more susceptible to neuromuscular fatigue which can negatively impact their activities of daily living and work productivity (Fritschi and Quinn, 2010; Goedendorp et al., 2014; Senefeld et al., 2018). Muscle fatigue, defined as an exercise-induced reduction in the ability of muscles to produce desired force or power (Enoka and Duchateau, 2008), can increase the risk of musculoskeletal disorders (MSDs; Gallagher and Schall, 2017). In order to mitigate the risk of MSDs in diabetic individuals, the mechanisms behind the effect of diabetes on neuromuscular performance need to be well understood first. Thus, the impact of this disease on neuromuscular fatigue and associated central and neural mechanisms need to be investigated.

Individuals with Type 1 Diabetes (T1D) exhibit both higher fatigability and lower muscle strength. For example, T1D is associated with reduced muscle fiber volume and reduced maximal isometric and isotonic force production (Krause et al., 2011). Adults with T1D have also exhibited slower motor unit discharge frequency or the rate at which motor neurons activate the muscle fibers and fatigued ∼45% sooner than their healthy counterparts while performing fatiguing sub-maximal contractions of the knee extensor muscles, implicating the role of central fatigue (Almeida et al., 2008). T1D patients with polyneuropathy have also shown lower muscle strength than healthy controls for similar submaximal contractions of the knee extensor muscles (Orlando et al., 2017). However, whether the aforementioned neuromuscular effects of T1D are similar on upper extremity musculature, particularly hand/arm muscles, remains unknown.

Chronic diseases, such as diabetes and obesity, are also associated with changes in brain structure, reductions in brain volume, and altered gray and white matter integrity (Pannacciulli et al., 2006; Willeumier et al., 2011; Kullmann et al., 2012; Biessels and Reijmer, 2014). In particular, T1D is associated with decreased gray matter volume in the frontal, posterior, and temporal cortex, decreased fractional anisotropy (degree of restriction of fluid along its axes) in posterior regions of the brain and, altered functional connectivity in several brain regions (Van Duinkerken et al., 2012; Biessels and Reijmer, 2014). Children with T1D exhibit higher functional connectivity for cognitive work to compensate for the negative influence of the disease on the brain structure (Mazaika et al., 2020). However, no studies have investigated if there is a similar compensatory increase in functional connectivity due to T1D during motor tasks in adults.

Neuromuscular fatigue is an extreme perturbation to the motor system, resulting in altered neural drive (Racinais et al., 2007), and loss of functional connectivity in motor-function related brain regions (Peltier et al., 2005). With chronic diseases, increased fatigability may be driven by neural impairments. For example, in obese adults, greater motor impairments have shown to be accompanied with lower frontal lobe activation (Mehta and Shortz, 2014; Osofundiya et al., 2016) and reduced functional connectivity in the motor areas of the brain (Rhee and Mehta, 2018). Thus, it is possible that neural signatures of fatigue are different for T1D than healthy adults, owing to the disease-related changes in brain structure described earlier. Indeed, a prior examination of motor unit discharge frequency during fatiguing exercises have implicated the role of central mechanisms in adults with T1D (Almeida et al., 2008). Thus, further investigations on how neural signatures differ with T1D may potentially shed some light on supraspinal contributions of neuromuscular fatigue in this population.

Neuromuscular strength and fatigue mechanisms are also sex-dependent. While males exhibit more strength, they tend to be more fatigable than females when performing isometric contractions at the same intensity (Hunter and Enoka, 2001; Enoka and Duchateau, 2008). Sex differences in fatigability are commonly associated with a difference in size and volume of muscle fibers and absolute contraction intensity of the muscles (Enoka and Duchateau, 2008; Hunter, 2017). Men and women have shown different performance strategies for the same motor task (Hunter, 2014). While males tend to have stronger within-hemispheric connectivity, females have stronger interhemispheric connectivity (Ingalhalikar et al., 2014; Rhee and Mehta, 2018). These sex differences in the neural strategies of motor performance suggest that T1D may affect motor performance of males and females differentially. However, to our knowledge, such difference have not been studied.

To address the gaps identified, the aim of this study was to determine the impact of T1D on handgrip fatigue and motor performance strategies and examine associated sex differences, neural activation patterns, and functional connectivity. Functional hemodynamic responses from motor-function related brain regions [i.e., supplementary motor area (SMA), premotor area, and the primary motor cortex] were monitored using functional near-infrared spectroscopy (fNIRS) among a sample of T1D and healthy adults, along with strength and motor performance data, during fatiguing submaximal isometric handgrip contractions. To measure motor performance strategies, covariance of force and approximate entropy (ApEn) were measured. Covariance of force is a measure of force fluctuations, and is correlated with motor unit firing behavior and captures physiological changes with fatigue (Contessa et al., 2009). ApEn was used as a measure of force complexity to indicate the adaptability of the neuromuscular system to fatiguing contractions (Pethick et al., 2015). Based on prior evidence on T1D-related lower extremity motor function impairments (Almeida et al., 2008), we hypothesized that individuals with T1D will have lower initial handgrip strength and higher handgrip fatigability as compared to healthy controls, and that this relationship will be sex-dependent. Given that T1D is associated with structural brain changes (Biessels and Reijmer, 2014), we also hypothesized that these group-dependent neuromuscular outcomes will be associated with different neural activation and functional connectivity patterns.

## Materials and Methods

### Participants

Forty-two adults, twenty with T1D (9 M, 11 F) and twenty-two healthy controls (10 M, 12 F), with no accompanying comorbidities were recruited from the local community (Table 1). All participants were over 18 years of age, right-hand dominant, and without any known cardiovascular diseases or musculoskeletal injuries in their dominant hand. All participants self-reported to be sedentary or recreationally active. The study was approved by the Institutional Review Board of Texas A&M University and written informed consent was collected from all participants before data collection.

**TABLE 1:** Participant demographics.

	Healthy		T1D	
	Female (*n* = 12)	Male (*n* = 10)	Female (*n* = 11)	Male (*n* = 9)
Age (years)	22 ± 2.66	24.9 ± 5.26	18.91 ± 0.94	26 ± 10.38
Height (m)	1.66 ± 0.06	1.75 ± 0.06	1.66 ± 0.06	1.77 ± 0.06
Weight (kg)	64.8 ± 13.6	73.76 ± 11.92	67.87 ± 13.12	75.9 ± 19.65
BMI (kg/m^2^)	23.26 ± 3.47	24.07 ± 3.16	24.7 ± 5.44	24.1 ± 4.39

*All measures presented as mean ± SD.*

### Procedures

**FIGURE 1:**
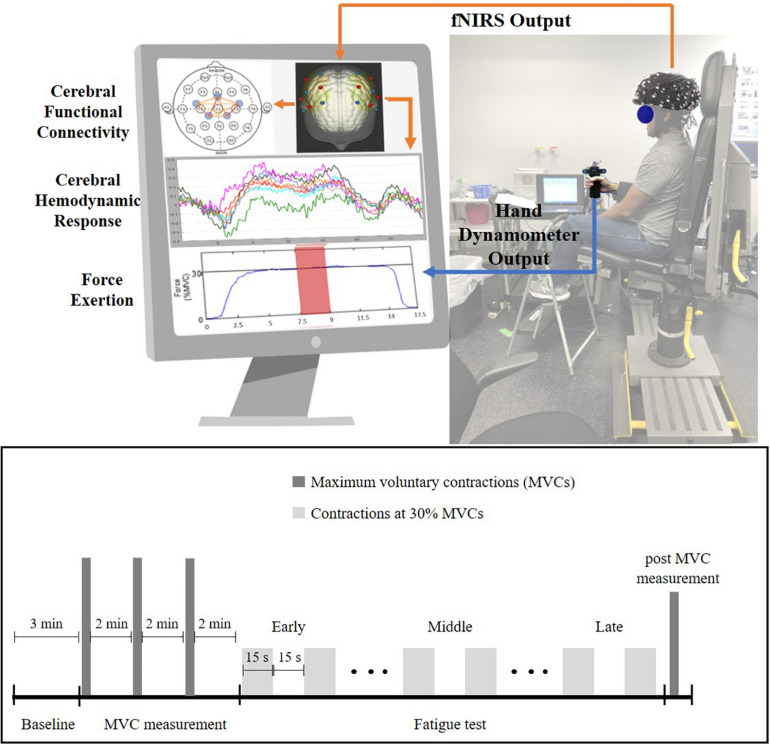
Experiment setup (top) and experiment protocol (bottom) for the handgrip fatigue experiment. Early phase is the first one-third of the 30% MVC period, Middle phase is middle one-third and Late phase is last-one third of the 30% MVC period. Force data and fNIRS data were recorded throughout the experiment. Participant had real-time feedback of their handgrip performance through a display monitor.

After providing consent, participants were instrumented with several sensors, including an fNIRS headcap (NIRx Medical Technologies, New York, NY, United States) and surface electromyography (EMG) sensors (Delsys Incorporated, MA, United States) on the forearm muscles. Specific descriptions of the EMG data acquisition processes and subsequent analysis and interpretation will be a focus elsewhere and are not discussed here. After bioinstrumentation, participants underwent a 3-min baseline period, where they were asked to close their eyes and relax in a quiet dark room. Following this, participants were seated upright with their right elbow at 90° and their lower arm resting on an armrest holding a hand dynamometer (Biopac, CA, United States, Figure 1). Participants performed isometric maximum voluntary contractions (MVCs) three times with 2-min rest between each trial (these tasks are referred to as pre-MVCs). Then, the maximum of the three MVCs was recorded as initial strength and utilized to determine a target line set at 30% of initial strength. The fatigue test required participants to perform repeated submaximal contractions at 30% of initial strength values. These contractions were sustained for 15 s followed by 15 s of rest (Figure 1) till voluntary fatigue or inability of maintaining contractions at the target level of 30% MVC. Participants could see their performance on a computer screen throughout the experiment and they were instructed to maintain their force at the target level as accurately and for as long as they could (Figure 1). Once participants determined they couldn’t hold the force any longer, they performed an additional MVC that collected post-MVC value immediately after the termination of submaximal contractions.

### Measurements

#### Strength, Fatigue, and Motor Variability

Initial strength, strength loss, and endurance times were recorded for all participants. Initial strength was determined as the maximum value of the pre-MVCs. Strength loss (represented as % of initial strength) was calculated from the post-MVC taken after the termination of the fatiguing exercise. Endurance time was defined as the duration between the start of submaximal contractions and when the task was terminated due to fatigue. Handgrip force was recorded using the hand dynamometer (Biopac, CA, United States) at 1000 Hz and the force data were filtered through a low pass Butterworth filter at 15 Hz. The middle 10 s of each 15 s contraction period were extracted from the force data. To measure changes in force variability with fatigue, coefficient of variation (CV) of the extracted force data were measured. Force CV was calculated as Standard deviation (SD)/mean (Duan et al., 2018; Zhu et al., 2020). ApEn of force data were used to measure changes in motor complexity with fatigue (Pethick et al., 2015). ApEn  measures the apparent randomness in time-series data. ApEn (*m*, *r*, and *N*) is the average of the negative natural logarithm of the conditional probability that a series of data (or template) of length m is repeated during the time series of length *N* with *r* as the tolerance for accepting matches. Based on previous literature, *r* has been recommended to be between 0.1 × SD and 0.25 × SD of the time series signal (Richman and Moorman, 2000), for this study 0.1 was selected. The length of the data sequences to be compared denoted as m, was set at *m* = 2 based on previous literature (Forrest et al., 2014; Pethick et al., 2015). ApEn was calculated using Eq. (1). *B*_*i*_ is the number of matches to the *i*th template of length *m* and *A*_*i*_ is the number of those matches that remain similar for the m + 1th point for the *i*th template. Based on previous studies (Forrest et al., 2014; Pethick et al., 2015), the parameters were set as *m* = 2 and *r* = 0.1 × SD. Both force CV and force ApEn were calculated for each contraction. These values were then averaged for the Early, Middle and Late phase of the fatigue test for each participant.

(1) $$\mathrm{ApEn}\,\left(m,r,N\right)=\frac{1}{N-m}\,\sum_{i=1}^{N-m}\log\frac{A_{i}}{B_{i}}$$

#### Brain Activation and Functional Connectivity

**FIGURE 2:**
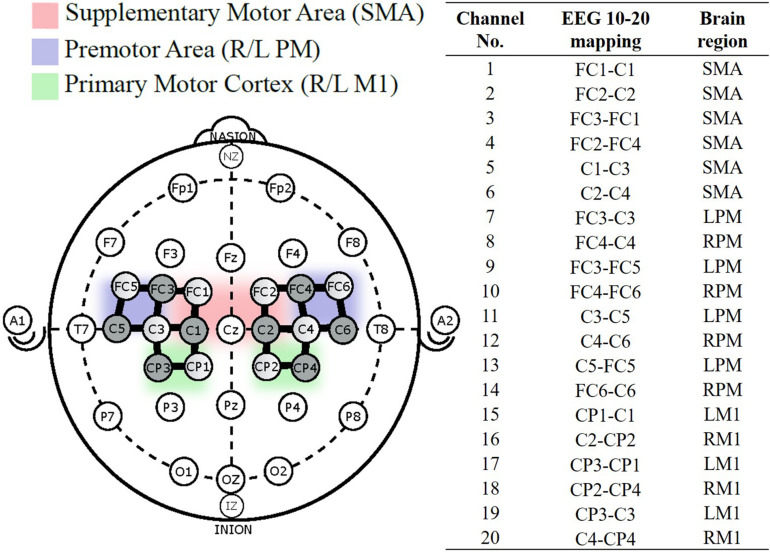
fNIRS probe placement based on the international 10–20 system, with 8-sources (shaded), 8-detectors (unshaded), and 20 channels (solid black line) between them. Channel numbered 1–20 were divided into five regions of interest (Cao et al., 2015).

Hemodynamic responses were obtained using the continuous wave fNIRS system (NIRx Medical Technologies, NY, United States) that utilizes two wavelengths of infra-red light (between 650 nm and 900 nm). The fNIRS probe design with 8 emitters and 8 detectors was positioned based on the international 10/20 EEG system using a probe design software (NIRSITE^TM^, NIRx Medical Technology, NY, United States) and 20 channels were obtained (Figure 2). The probe format was designed to cover motor regions involved in fatiguing contractions based on previous literature (Rhee and Mehta, 2018, 2019). A MATLAB (MathWorks, MA, United States) based fNIRS signal processing software (Homer 2, https://homer-fnirs.org/) was first used to transform the light intensities to optical density by taking the logarithm of the input signal. The optical density was then low pass filtered at a frequency of 3 Hz based on a previous study (Rhee and Mehta, 2018). However, when the results from the low pass filtered data were compared to results without the low pass filtering, the results were same and this step was therefore, found redundant. Any possible motional artifacts were detected and corrected by using spline interpolation function, “hmrMotionCorrectSplineSG” in HOMER with parameter *p* = 0.99 (Scholkmann et al., 2010) and smoothed using a Kurtosis-based wavelet algorithm using the function “hmrMotionCorrectKurtosisWavelet” with kurtosis = 3.3 (Chiarelli et al., 2015) using predefined functions in Homer 2. Data from two participants were excluded from further analysis due to a large amount of motion artifacts.

To minimize physiological noise, data were band pass filtered between 0.01 Hz and 0.5 Hz (Zhu et al., 2019). The Modified Beer-Lambert law was then used to convert these signals to oxygenated hemoglobin (ρHbO) and deoxygenated (ρHbR) hemoglobin concentrations. ρHbO was selected for this study because it is more sensitive to cerebral hemodynamic changes compared to ρHbR (Hoshi et al., 2001). Task related brain activations were derived by averaging 2 s ρHbO signal around the maximum within each trial, a method that has been used in the investigation of brain activation during motor tasks (Obrig et al., 1996; Hyodo et al., 2016; Vrana et al., 2016; Zhu et al., 2019).

**FIGURE 3:**
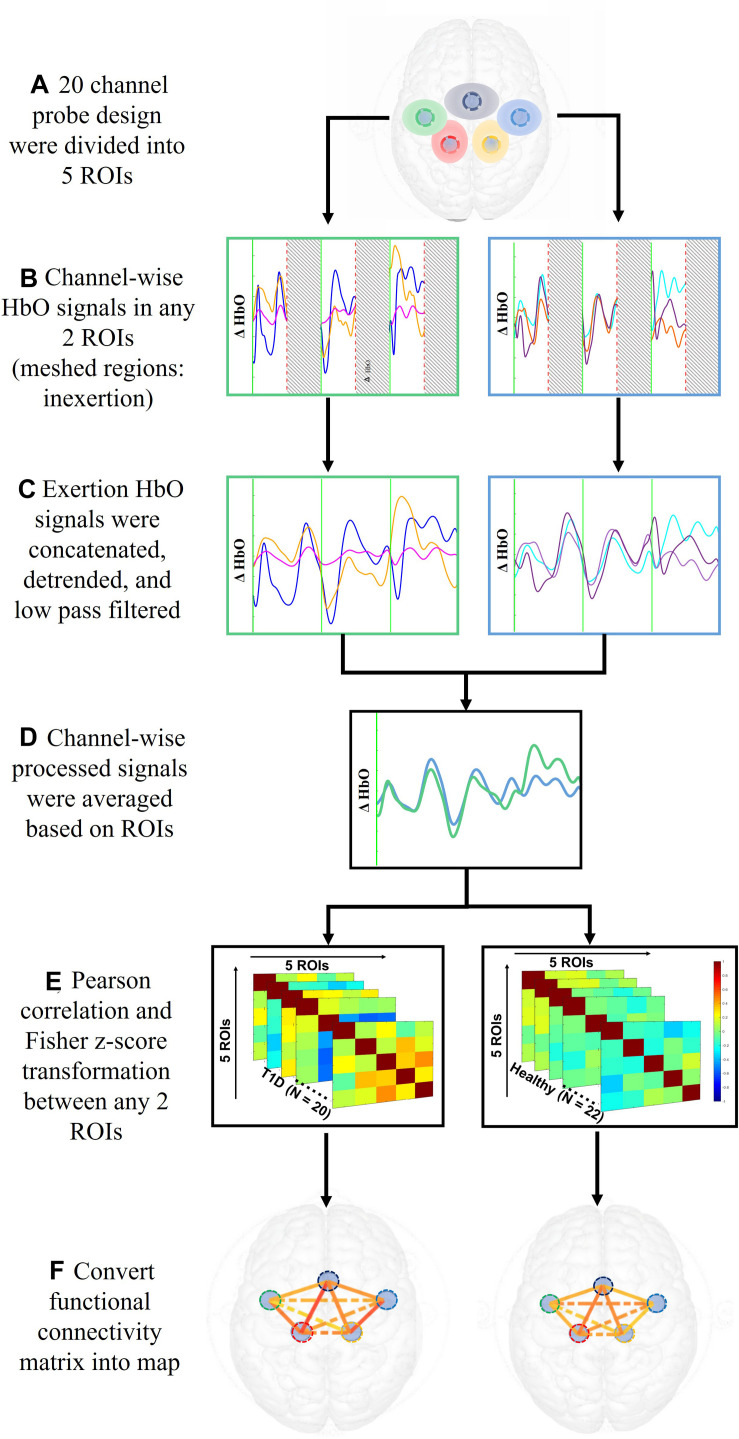
Data analysis steps performed on ρHbO signals from the period of fatigue test to the calculation of functional connectivity.

The 20 channels were averaged into five regions of interest (ROIs; Cao et al., 2015) – the motor area (SMA), right and left premotor area (R/L PM), and the right and left primary motor cortex (R/L M1). Figure 3 illustrates the process to compute the functional connectivity between the ROIs. First, the averaged ρHbO signals during the hand force contractions were extracted for the early and late phases separately. The signals within each phase were concatenated, detrended and low pass filtered at 0.1 Hz (Rhee and Mehta, 2018). To measure connectivity between regions, Pearson correlations (*r*_*p*_) across all ROIs were calculated and then converted to Fisher’s *Z*score using Eq. (2). Regions with absolute value of Fisher *Z*score greater than 0.3 between them were considered functionally connected and this threshold was selected based on prior literature (Hocke et al., 2016).

(2) $$\mathrm{Fisher}\,\mathrm{Zscore}=0.5\times \ln\frac{1+r_{p}}{1-r_{p}}$$

### Statistical Analysis

To check for goodness of fit of the data to a normal distribution, the Anderson-Darling test was performed on initial strength, strength loss, endurance time, CV force, ApEn and peak activation of each channel at a threshold *p* = 0.05 for each subgroup (healthy males and females, T1D males and females). After confirming goodness of fit, separate Sex (male, female) × Condition (T1D, healthy) Analyses of Variance (ANOVAs) were performed on initial strength, strength loss, and endurance times. Additionally, separate Phase (Early, Middle, Late) × Sex (male, female) × Condition (T1D, healthy) repeated measures ANOVAs were performed on the peak amplitude of ρHbO from each fNIRS channel seperately, CV force, and ApEn to measure changes in neural activation, force variability and complexity. The Benjamin Hochberg procedure for false discovery rate corrections was applied to determine significance at level *q* = 0.10 (Benjamini and Hochberg, 1995) for the fNIRS analysis to account for multiple comparisons. Independent *t*-tests were performed on the Fisher *Z*scores of functionally connected regions between healthy and T1D populations, separated by sex (male, female) and phase (early, late), at significance level of α = 0.05. In *post hoc* analysis, interaction effects at a significance level of α = 0.05 were reexamined using one-tailed pairwise *t*-tests.

## Results

### Strength and Fatigue

Table 2 shows the fatigue and strength parameters for the four groups (healthy males and females, T1D males and females). All groups exhibited a strength loss between 36% and 47% of their initial strength, indicating that all groups were similarly fatigued. Females exhibited lower initial strength [*F*(1,38) = 20.492, *p* < 0.0001, and η^2^ = 0.304] and greater strength loss [*F*(1,38) = 5.6275, *p* = 0.023, and η^2^ = 0.127] as compared to males (Table 2). Males and females had similar endurance times (*p* = 0.264). Participants with T1D had significantly lower initial strength as compared to healthy controls [*F*(1,38) = 6.983, *p* = 0.012, and η^2^ = 0.104]. However, there was no effect of condition on endurance time (*p* = 0.206) or strength loss (*p* = 0.395) of participants. No sex × condition interaction effects were observed for initial strength, endurance time, or strength loss (all *p*’s > 0.196).

**TABLE 2:** Fatigue and strength measures for all participants.

	Healthy		T1D	
	Female (*n* = 12)	Male (*n* = 10)	Female (*n* = 11)	Male (*n* = 9)
Initial strength (Kg)	7.23 ± 1.41	12.16 ± 4.11	6.11 ± 1.48	8.82 ± 3.27
Strength loss (% Initial Strength)	45% ± 11%	36% ± 11%	47% ± 11%	39% ± 14%
Endurance time (s)	1722.19 ± 1426.93	2219.48 ± 1048.5	1451.26 ± 599.57	1673.68 ± 693.67

*All measures presented as mean ± SD.*

### Motor Variability

There was a main effect of fatigue on force CV [*F*(2,33) = 15.491, *p* < 0.0001]. *Post hoc* analysis revealed that force CV increased from 0.064 ± 0.040% in the Early phase to 0.069 ± 0.034% (Middle) to 0.144 ± 0.087% in the Late phase. A main effect of fatigue was also observed on force ApEn [*F*(2,35) = 9.7817, *p* = 0.0004]. *Post hoc* analysis revealed that ApEn decreased with fatigue from 0.031 ± 0.009 (Early) to 0.027 ± 0.011 (Middle) to 0.023 ± 0.011 (Late). There was no main effect of sex on force CV (*p* = 0.195) or force ApEn (*p* = 0.7603). A significant main effect of condition [*F*(1,36) = 4.6524, *p* = 0.0378] was found on force ApEn, where adults with T1D exhibited lower force ApEn 0.025 (0.009) as compared to healthy adults 0.030 (0.010). No condition effect was observed on force CV (*p* = 0.446). Additionally, no 2-way or 3-way interaction effects between phase, sex, and condition were observed for force CV or ApEn (all *p*’s > 0.0872).

### Brain Activation

**FIGURE 4:**
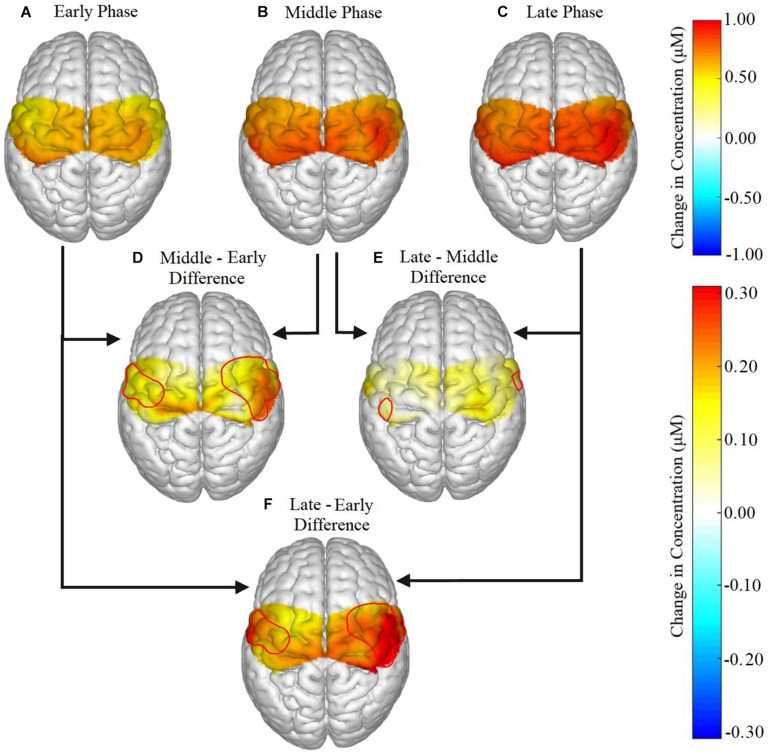
Brain activation for all participants in the **(A)** early, **(B)** middle, and **(C)** late phases, and the difference in activation between each phase represented as **(D)** middle – early, **(E)** late – middle, **(F)** late – early. Significant phase effects were found for channels 2, 4, 6, 8, 12, 14, and 18 in the ipsilateral hemisphere and in channels 5, 7, 9, and 11 for the contralateral hemisphere for **(D)** middle – early and **(F)** late – early. For **(E)** late – middle, the only significant differences were found in channels 14 and 11.

**FIGURE 5:**
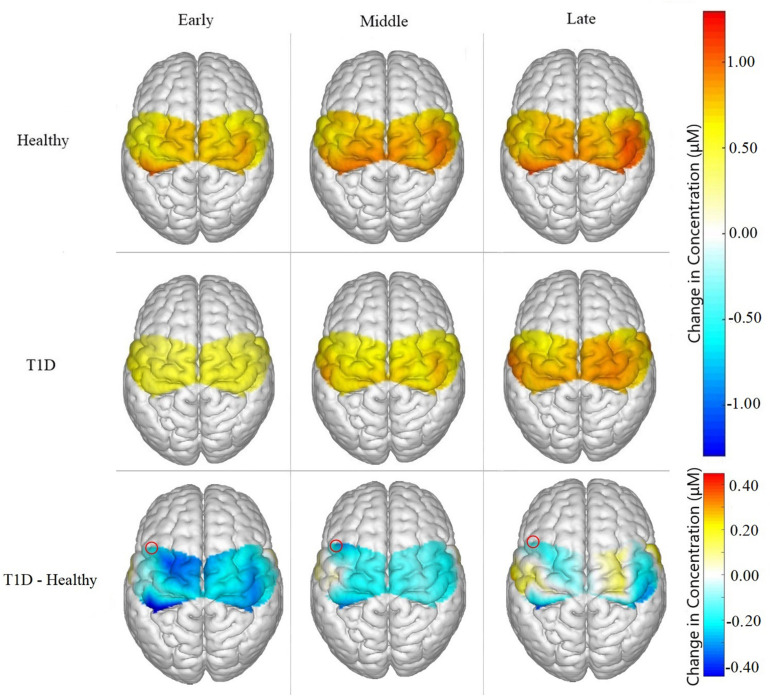
Brain activation for Healthy participants, T1D participants, and the difference between them (T1D – Healthy) for Early, Middle and Late phases. T1D participants showed significantly lower neural activation than the healthy controls in the early phase (*p* = 0.035) and marginally lower activation in the middle phase (*p* = 0.0626). A marginal main effect of condition was observed in channel 3, highlighted in the figure above, of the SMA (*p* = 0.077).

Table 3 lists the fNIRS channels that showed a significant main effect of phase on neural activation. In general, fatigue progression was associated with increasing neural activation in the channels listed in Table 3. Figure 4 shows the contrast maps for brain activation in the three phases for all participants and the difference in activation between the phases. A main effect of sex was found: males exhibited significantly greater neural activation than females in the SMA [channel 3: *F*(1,26) = 4.455, *p* = 0.045; channel 5: *F*(1,25) = 6.309, *p* = 0.019]. *Post hoc* analysis revealed that for channel 3, activation for males was significantly higher than females for the Middle (*p* = 0.0294) and Late phase (*p* = 0.0332) and marginally higher for the Early phase (*p* = 0.0629). For channel 5, males had significantly higher activation for the Early (*p* = 0.0081), Middle (*p* = 0.0077), and Late phase (*p* = 0.0461). A marginal main effect of condition (Healthy > T1D) was observed in the channel 3 of the SMA [Figure 5; *F*(1,26) = 3.383, *p* = 0.077]. To further investigate the effect of T1D on activation in channel 3, paired *t*-tests were formed between T1D and healthy adults for Early, Middle and late phases. It was found that T1D adults exhibited significantly lower activation than the healthy controls in the early phase (*p* = 0.035) and marginally lower activation in the middle phase (*p* = 0.0626). Additionally, paired *t*-tests between the Early, Middle and Late phase activation for T1D and healthy adults were performed that showed a significantly higher activation in the late phase from the early phase only for T1D adults (*p* = 0.007). A phase × gender effect was observed where increased activation in the RPM with fatigue was significantly higher for males than females [channel 14: *F*(2,27) = 6.372, *p* = 0.0054]. Finally, a Phase × Sex × Condition interaction effect was found in the SMA [channel 4: *F*(2,29) = 3.473, *p* = 0.044]. *Post hoc* analysis revealed that while increased activation was observed in both T1D females (*p* = 0.016) and healthy males (*p* = 0.024) from the Early to Middle phase, this was not observed in healthy females or T1D males.

**TABLE 3:** Channels where significant phase effect was observed with False Discovery Rate correction applied at threshold *q* = 0.10.

Channel	Region of interest	*p*-value	*F*-statistic	*Post hoc* comparisons
2	SMA	0.017	*F*(2,26) = 4,788	Early < Middle, Late
4	SMA	0.012	*F*(2,29) = 5.125	Early < Middle, Late
5	SMA	0.001	*F*(2,24) = 9.173	Early < Middle, Late
6	SMA	0.007	*F*(2,25) = 6.169	Early < Middle, Late
7	LPM	0.004	*F*(2,27) = 6.824	Early < Middle, Late
8	RPM	0.0002	*F*(2,31) = 11.242	Early < Middle, Late
9	LPM	0.001	*F*(2,25) = 9.000	Early < Middle, Late
11	LPM	0.006	*F*(2,28) = 6.141	Early < Middle < Late
12	RPM	<0.001	*F*(2,28) = 15.081	Early < Middle, Late
14	RPM	<0.0001	*F*(2,27) = 23.548	Early < Middle < Late
18	RM1	0.016	*F*(2,27) = 4.836	Early < Middle, Late

*For *post hoc*, paired *t*-tests were performed between activation for Early and Middle and between Middle and Late for each channel. Activation increased in all channels from Early to Middle phase. Activation increased for channels 9 and 20 from the Middle to Late Phase.*

### Functional Connectivity

**FIGURE 6:**
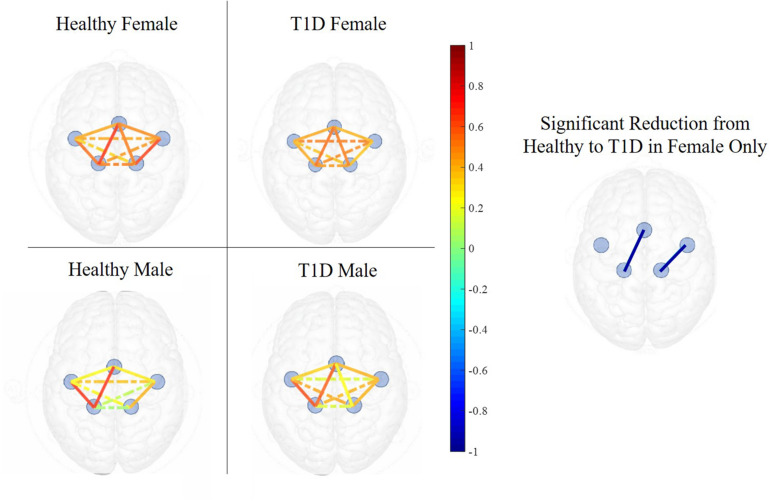
Functional connectivity maps of Healthy (left) and T1D (right) males and females during the early phase of hand-grip motor task. The color of each edge depicts the Pearson correlation between ROIs. Nodes connected with solid lines show intra-hemispheric connectivity and nodes connected with dotted lines show inter-hemispheric connectivity. Healthy females exhibited higher intra-hemispheric functional connectivity between the SMA and LM1 and between RPM and RM1 in the early phase. There was no significant difference between the connectivity of healthy and T1D males.

Functional connectivity reduced from early to late phase between LPM and RPM (*p* = 0.017), LPM and LM1 (*p* = 0.004), LPM and RM1 (*p* = 0.008), LPM and SMA (*p* = 0.029), RPM and RM1 (*p* = 0.025), LM1 and RM1 (*p* = 0.008), LM1 and SMA (*p* = 0.007), and SMA and RM1 (*p* = 0.001). Females with T1D exhibited significantly lower functional connectivity between SMA and LM1 (*p* = 0.045) in the Early phase as compared to their healthy counterparts (Figure 6). However, similar condition effects were not observed in the Late phase in females. Additionally, there were no differences in connectivity patterns between healthy and T1D males in both Early and Late phases (all *p*’s > 0.187).

## Discussion

The present study investigated the impact of T1D on handgrip fatigue, neural activation, functional connectivity and associated sex differences. The key findings from this study were: (1) T1D adults exhibited lower initial strength as compared to their healthy counterparts, however, fatiguability remained comparable across the two groups; (2) T1D was associated with lower force complexity (i.e., lower approximate entropy of force) as compared to healthy controls; (3) lower force complexity with T1D was accompanied with greater activation of the SMA from early to late fatigue phase, and; (4) T1D females showed reduced connectivity of the motor function related cortical regions than their healthy counterparts. Collectively, these results indicate that, in comparison to healthy adults, T1D adults exhibit different motor and neural strategies to preserve motor performance, and that these strategies are sex-dependent.

### Effect of T1D on Handgrip Strength and Fatigue

As hypothesized, T1D adults demonstrated lower initial strength as compared to their healthy counterparts. These results are similar to previous studies that have reported lower knee extensor strength for T1D populations (Orlando et al., 2017). Whereas previous studies have reported lower endurance time for T1D populations (Almeida et al., 2008; Allen et al., 2015; Orlando et al., 2017), the present study found comparable endurance times and strength loss between the healthy and T1D groups. It is likely that the discrepancy in the literature may be driven by the type of muscle group being tested. The previous studies examined lower extremity muscle groups while the present study targeted fatigue evaluation of upper extremity hand-arm muscles during an intermittent handgrip test. In T1D, muscle groups with higher proportion of fatigue resistant type 1 fibers are known to exhibit smaller decline in endurance time and hand-arm muscles are known to have a higher proportion of type 1 fibers than quadriceps muscles (Andersen, 1998; White et al., 2013). This may potentially explain the similar endurance times observed between T1D and healthy adults in the present study. Future work is warranted to examine if neuromuscular fatigability in T1D adults is muscle-dependent.

### Effect of T1D on Motor Variability Measures

Force ApEn, which is a measure of force complexity, was found to be lower for T1D and decreased over time with the onset of fatigue for both the healthy and T1D groups. Loss of force complexity reflects a decreased flexibility in force control and reduced adaptability to external perturbations to the motor system (Lipsitz and Goldberger, 1992; Li et al., 2013; Pethick et al., 2015). Thus, lower force complexity in T1D observed indicated an impaired adaptability to motor tasks under perturbing conditions like neuromuscular fatigue (Pethick et al., 2015; Suda et al., 2017). Force complexity also depends on visuomotor corrections based on visual feedback of motor performance and the neural mechanisms associated with visuomotor processing (Li et al., 2013). In this study, participants were provided a visual feedback of their performance to enable them to trace their force output at 30% MVC. Hence, lower ApEn in T1D is also indicative of lower adaptability to visuomotor correction. However, TID participants were still able to maintain similar levels of performance to healthy controls as reflected by both endurance times and strength loss. This suggests that the impairment in the ability to adapt to motor tasks with fatigue was compensated elsewhere.

### Effect of T1D on Brain Activation

Type 1 diabetes adults exhibited significantly lower activation in the SMA in the Early phase but activation comparable to healthy adults in the late phase. Activation of SMA is associated with motor planning and performance of complex motor tasks (Tanji, 1994; Gerloff et al., 1997). Involvement of SMA in performance of motor tasks is also known to increase with motor task complexity (Shibasaki et al., 1993). This indicates that perceived complexity of motor tasks increased with fatigue for T1D participants as initially, T1D had lower activation in the SMA, however, the perturbing effect of fatigue caused an increase in the SMA activation so that it became comparable to healthy controls in the late phase. Therefore, the increases in activation of the SMA with fatigue could be a compensatory response to the decreased flexibility in force control (as observed with lower ApEn) in T1D adults. The onset of T1D, which causes structural and functional changes in the brain, most commonly occurs in childhood or adolescence years, which are also the developmental stages of the brain (Biessels and Reijmer, 2014). Mazaika et al. (2020) reported that T1D youth exhibit higher activation of the Rostro lateral Prefrontal Cortex (RLPFC) and the Supramarginal Gyrus (SMG) to compensate for the negative influence of T1D on cognitive performance. This is indicative of neural adaptation and brain development in youth with T1D in response to the inhibitory effects of the disease. In a similar vein, we propose that neural adaptation in individuals with T1D occurs in response to impaired motor adaptability. These results also highlight the importance of investigating both neural and motor performance signatures as they lead to better insights into the mechanisms of fatigue.

### Effect of T1D and Fatigue on Functional Connectivity

Fatigue development was accompanied by a decrease in functional connectivity between LM1 and other monitored ROIs. Declines in functional connectivity between RM1-SMA was also observed from Early to Late fatigue phase. These findings support previous studies that have also observed loss of functional connectivity with the onset of fatigue (Peltier et al., 2005; Rhee and Mehta, 2018). In the Early phase, T1D females had lower functional connectivity between LM1 and SMA than their healthy counterparts, while the connectivity resumed to be comparable to the healthy females in the late phase. Lower functional connectivity of the premotor and primary motor regions is indicative of impairments in motor planning and execution of movements (Hoshi and Tanji, 2007). However, the perturbing effect of neuromuscular fatigue is associated with strengthened functional connectivity between M1 and other associated motor regions (Jiang et al., 2012). These results therefore indicate that females with T1D may exhibit impaired neural connectivity which may hinder motor planning. In addition, it is possible that an acute fatiguing protocol may potentially alleviate such neural impairments.

### Limitations

First, the present study investigated the effect of T1D on neuromuscular fatigue of the hand-arm muscles during handgrip exercises. When compared to previous research, our results indicate that effect of T1D on neuromuscular fatigue may be muscle-dependent, which warrants further investigation. Second, this study observed different neural strategies in T1D, compared to healthy controls, for the performance of handgrip motor task. However, whether these neural strategies are an adaptive response to the influence of T1D in the developmental stages of the brain has not been conclusively proven. Third, as has been done for cognitive performance, future studies need to examine both the structural and functional changes with T1D to better understand neuromuscular fatigue mechanisms in this group. Fourth, blood glucose levels in the T1D adults were not collected in the present study which are otherwise typically monitored (Almeida et al., 2008; Orlando et al., 2017). Future studies are needed that expand on participant data collection with blood glucose levels to better interpret their impact on brain function. Fifth, in this study, the statistical results were FDR corrected at a significance level of *q* = 0.1 which is a more liberal statistical level for false discovery rate correction. Future work that will afford larger sample sizes and more focused regions of interests are planned to analyze neural responses using a more strict FDR correction. Finally, due to resource constraints of the probe design of fNIRS, activation of the prefrontal cortex (PFC) was not monitored. As functional connectivity between the PFC and motor areas has shown to be affected by fatigue in individuals with chronic diseases (Rhee and Mehta, 2018) and is also involved in motor tasks that require motor control based on visual feedback (Derosiere et al., 2014), future studies on T1D and fatigue may expand on other motor function related cortical regions, including the PFC.

## Conclusion

We investigated the effects of T1D on neuromuscular fatigue and associated neural signatures and examined potential sex differences. While fatigability between T1D adults and healthy controls were comparable, T1D adults exhibited lower force complexity and therefore, lower adaptability to motor task and fatigue. To compensate for this, T1D adults exhibited higher neural effort to maintain task performance. Sex differences were found on the impact of T1D on functional connectivity of motor regions, where only T1D females exhibited lower functional connectivity when compared to all other groups. These findings highlight two major implications. First, it is important to monitor both the neural and traditional fatigue measures, since one type of assessment alone does not provide a complete picture of the impact of fatigue in different groups. Second, that the T1D group exhibited different neural strategies to address fatigue development than healthy controls, highlights promise of developing group-specific intervention strategies for training, rehabilitation, and ergonomics considerations for individuals with chronic conditions.

## Data Availability Statement

The raw data supporting the conclusions of this article will be made available by the authors, without undue reservation.

## Ethics Statement

The studies involving human participants were reviewed and approved by Institutional Review Board of Texas A&M University. The patients/participants provided their written informed consent to participate in this study.

## Author Contributions

RM, FS, ME, and KQ conceptualized the study and edited the manuscript. RM supervised and guided the data collection. OT and YZ performed the data collection. OT, YZ, and CJ analyzed the data, synthesized the results, and drafted the manuscript. All authors contributed to the article and approved the submitted version.

## Conflict of Interest

The authors declare that the research was conducted in the absence of any commercial or financial relationships that could be construed as a potential conflict of interest.
